# Extensive Study on Hematological, Immunological, Inflammatory Markers, and Biochemical Profile to Identify the Risk Factors in COVID-19 Patients

**DOI:** 10.1155/2022/5735546

**Published:** 2022-04-21

**Authors:** Eman T. Ali, Azza Sajid Jabbar, Hadeel S. Al Ali, Saad Shaheen Hamadi, Majid S. Jabir, Salim Albukhaty

**Affiliations:** ^1^Department of Clinical Laboratory Sciences, College of Pharmacy, University of Basrah, Basrah, Iraq; ^2^Department of Pharmacology and Toxicology, College of Pharmacy, University of Basrah, Basrah, Iraq; ^3^Department of Physiology, Al-Zahraa College of Medicine, University of Basrah, Basrah, Iraq; ^4^Department of Internal Medicine, College of Medicine, University of Basrah, Basrah, Iraq; ^5^Applied Science Department, University of Technology, Baghdad, Iraq; ^6^Department of Chemistry, College of Science, University of Misan, Maysan 62001, Iraq

## Abstract

**Background:**

Tissue damage caused by COVID-19 could be detected by several clinical indicators including hematological, immunological, biochemical, and inflammatory markers. This study was to detect these clinical parameters to reveal the correlation between the factors and their roles in the development of COVID-19, to explore the hazard factors in severe cases.

**Materials and Methods:**

A total of 200 participants of both sexes were included in the study, with an age range of (25–72) years, categorized into three main groups: 50 healthy individuals, 62 mild infected patients, and 88 severe infected patients with pneumonia. Different hematological and clinical parameters were included in the analysis (Basrah city, Iraq). Serum levels of interleukin-6 (IL-6), ferritin, and high-sensitivity *C*-reactive protein (hs-CRP) were assessed for all participants using an enzyme-linked immunosorbent assay (ELISA). The liver, renal, and cardiac functions were assessed by clinical chemistry testing.

**Results:**

COVID-19 patients had leukocytosis, with an increased number of neutrophils and a decreased lymphocyte count, according to our findings. In regard to inflammatory parameters, both ESR and hs-CRP showed significant differences between the two groups, whereas IL-6 was significantly higher in the total severe group compared to the other two groups. Biochemical results revealed that each lactate dehydrogenase (LDH), ferritin, alanine aminotransferase (ALT), and aspartate aminotransferase (AST) had significant changes in the total severe group. Among pneumonic with an O2 requirement and pneumonic without an O2 requirement, there were significant differences in immunological and inflammatory markers (*p* > 0.05). The neutrophils-lymphocytes ratio (NLR) was highly elevated in severe who required O2. Moreover, IL-6, lymphocytes, and neutrophils were possible risk factors for COVID-19 infection, with the strongest influence of IL-6 with a high odds ratio (OR: 24.138, 95% CI: 8.437–30.65, *p* < 0.01). Furthermore, there were significant correlations among the indicators.

**Conclusion:**

Each of IL-6, lymphocytes, and neutrophils might represent major factors in the severity of COVID-19 and IL-6 plays the main role in inducing the inflammatory and pathophysiology process that is known as the cytokine storm.

## 1. Introduction

Coronavirus disease 2019 (COVID-19) is a global pandemic that first appeared and was reported in Wuhan city, China, in December 2019. It is caused by severe acute respiratory syndrome coronavirus 2 (SARS Co 2) [1, 2]. The virus invades the targeting organs such as the alveolar epithelial cells by binding the S1 domain of the viral spike protein to cellular receptor angiotensin-converting enzyme 2 (ACE2) [3]. The existence of ACE2 receptors in specific areas of the body may enable the virus to attack; this could be linked to COVID-19 infection attacks on the respiratory system or other organs and systems, resulting in aberrant biochemical profiles in COVID-19 patients. [4]. COVID-19 is usually asymptomatic or presents flu-like symptoms. On the other hand, it may complicate with a more serious course [5]. Several observational studies have shown that aberrance of immune-inflammatory response and development of cytokine storm might be the reasons behind multiorgan and dead ends of COVID-19 [6–9].

The effect of infection might last for several months even after a complete recovery [10]. According to what has been stated in previous immunological reports, the level of circulating inflammatory cytokines, mainly interleukin-6 (IL-6), was significantly elevated in severe cases [11–15]. Moreover, autopsy analyses have found that increased levels of the inflammatory markers were associated with necrosis and infiltrations of interstitial macrophages in each pulmonary, cardiac, and gastrointestinal tissue of COVID-19 patients [16]. T-lymphocytes are necessary to destroy viral infection; SARS CoV 2 dysregulates the immune response when it invades the host body [17–19]. Lymphopenia is a prominent clinical laboratory result abnormality that has been frequently detected in patients with severe COVID-19; in addition, SARS CoV 2 infection increases neutrophils and monocytes number [20–22]. However, mechanisms are likely mediated by inflammatory cytokine storm as a consequence of uncontrolled and dysfunctional immune response, evidenced by an increase in the serum levels of IL-6, lactate dehydrogenase (LDH), ferritin, C-reactive protein (CRP), D-dimer, myoglobin, and fibrinogen [23, 24]. Moreover, complete blood count- (CBC-) derived parameters and their relation to certain diseases have recently received attention from researchers. One of these CBC parameters is the neutrophil-to-lymphocyte ratio (NLR). NLR is considered to be a marker of inflammation and, due to its simplicity and low cost, has been studied in many medical conditions [25]. COVID-19 infection is related to an increased inflammatory burden. Indeed, neutrophil-to-lymphocyte ratio has been reported to be associated various inflammatory conditions including irritable bowel syndrome [26], Hashimoto's disease [25], type 2 DM [27], diabetic nephropathy [28], thyroid conditions [29], and even COVID-19 infection [30].

The objectives of this study are to assess routine hematological, immunological, inflammatory markers, and clinical biochemistry test profile changes among individuals who had tested positive following COVID-19 real-time reverse transcription-polymerase chain reaction (rRT-PCR) laboratory tests, to reveal the correlation between the factors adopted in the study and their relationship to the disease form and to explore the major factors that are responsible for the development of COVD-19 into severe as an attempt for more understanding of this novel virus to guide preventive and curative interventions.

## 2. Material and Methods

### 2.1. Study Design

This cross-sectional study was performed on COVID-19 patients, in Basrah city, Iraq. The patients were admitted for the period from October 2020 to August 2021. Detection and diagnosis of the disease were based on clinical manifestations and laboratory test results according to the World Health Organization interim guidelines [31].

### 2.2. Patients

The participants in the study were 200 individuals of both sexes, with an age range of 25–72 years. They were categorized into three main groups: 50 healthy individuals as a control group (19 females and 31 males) and 62 patients with mild COVID-19 (24 females and 38 males), manifested by mild signs and symptoms without evidence of pneumonia.

All clinical investigations were undertaken for mild patients within the range of the incubation period (1–14) days and during the early phase of COVID-19. The clinical features of the disease typically appear around (5-6) days of incubation period after exposure. The most frequent signs were fever, cough, fatigue with the possibility of production of sputum, and headache, and another sign recorded in several cases was diarrhea. Furthermore, some cases of patients presented with a loss of sense of smell or taste. The third group included 88 severe infected patients (28 females and 60 males) with pneumonia. This group was further categorized into two subgroups: 34 severe patients with pneumonia but without the requirement for oxygen and 54 severe patients with pneumonia and requirement for oxygen (respiratory rate <breathes/min., O2 saturation >93%). The most severe patients who definitely required mechanical ventilator support due to respiratory failure, who were admitted to an intensive care unit (ICU) as a result of multisystem organ failure, or patients who presented to private laboratories with shock were deemed serious cases and excluded from this study. Blood count and biochemistry findings were investigated in the period from 7 to 14 days from initial symptoms onset. There is a surge in the clinical presentation of COVID-19 with pronounced increases in the levels of inflammatory mediators and proinflammatory cytokines that result in cytokines storm. All patients were well-investigated and they could answer a detailed form of a questionnaire. Clinical and epidemiological data of all confirmed COVID-19 patients were collected in a private clinical laboratory and consulting clinic. Independent doctors reviewed and verified all information precisely to make sure any information was accurate. [32]. The severity of COVID-19 was determined following Chinese guidelines (version 7.0).

### 2.3. Exclusion Criteria

Pregnancy, obesity, comorbidities, chronic airway diseases, chronic renal patients on dialysis, patients with autoimmune diseases, and patients on chemotherapy were excluded from the study.

### 2.4. Questionnaire

Required fundamental information was obtained from all participants in this study by a detailed form of a questionnaire that included demographic characteristics (gender and age), personal lifestyle (cigarette smoking and alcohol consumption), and health status (medications taken, genetic diseases in addition to other diseases like blood diseases, hypertension, diabetes mellitus, and renal diseases).

## 3. Laboratory Procedures

### 3.1. The Imaging Data

In all patients, infection with SARS COV 2 was confirmed positive by real-time reverse transcription-polymerase chain reaction (rRT-PCR) assay detection of virus-specific nucleic acid in sputum and throat swab specimens, collected from the upper respiratory system of patients by the guidelines for collection. Chest radiography and computed tomography (CT) scans with high resolution were performed for all patients in the initial days after admission to a private clinic. The imaging data were reviewed and interpreted by two consultant radiologists. A computed tomography scoring system was used to assess the extent of the disease.

### 3.2. Sample Collection

Around 5 ml of venous blood was drawn from each COVID-19 patient and healthy individual enrolled in this study after obtaining written consent from all participants using the usual method; then, a blood sample was immediately poured into two different laboratory tubes. The rest of the whole blood was collected in a serum separating gel tube to obtain blood serum for further laboratory tests. Collected samples were kept cold in a wet icebox until testing to avoid the effect of variable temperatures. All laboratory tests for blood samples were carried out on the same day of collection and in a private medical laboratory.

### 3.3. Clinical Chemistry Test

The liver, renal, and cardiac functions were assessed using routine clinical chemistry testing. The liver function tests included alanine aminotransferase (ALT), aspartate aminotransferase (AST), total bilirubin, direct (conjugated) bilirubin, and albumin, whereas the cardiac function tests comprised Troponin T, LDH, D-dimer, and ALT. Furthermore, urea and creatinine were stated as indicators for renal function tests, and the concentration of glucose, as well as laboratory tests, was determined depending on the unique medical condition of COVID-19 cases. Blood serum samples were processed and analyzed on a fully automated clinical chemistry analyzer (Abbott, Architect, USA).

### 3.4. Hematological Profile

2 ml of blood in a test tube, with ethylenediaminetetraacetic acid (EDTA) anticoagulant, was used for measurement of the vital hematological parameters. A blood count test was performed to measure hematological parameters using an automated hematology analyzer (Ruby, Germany). The following hematological parameters were determined: white blood cells (WBC) count and their subpopulations (neutrophils, monocytes, and lymphocytes), red blood cells (RBC) count, and hemoglobin (Hb) concentration. According to the reference values of the WHO, patients with hemoglobin levels <12.0 g/dl in adult women and <13.0 g/dL in adult men were diagnosed as anemic. The neutrophil-lymphocyte ratio (NLR) was computed by dividing the total number of neutrophils by the total number of lymphocytes.

### 3.5. Erythrocyte Sedimentation Rate Test

The erythrocyte sedimentation rate (ESR) was measured using the Westergren method as a nonspecific screening test. The findings of the ESR test are measured in millimeters per hour (mm/hr) [33].

### 3.6. Enzyme-Linked Immunosorbent Assay

#### 3.6.1. Determination of Interleukin-6 Cytokines

The interleukin-6 level was estimated using an enzyme-linked immunosorbent assay (ELISA), and serum IL-6 levels were determined for all patients with and without lymphopenia and controlled following the manufacturer's instructions and guidelines (PeproTech, USA). The normal reference value for IL-6 was 0–7 pg/ml.

#### 3.6.2. Evaluation of High-Sensitivity C-Reactive Protein Test

A high-sensitivity C-reactive protein (hs-CRP) test can detect the level of serum CRP which is a clinical marker of inflammation. CRP concentration was measured in the serum of total COVID-19 patients (with and without lymphopenia) and healthy control individuals using the ELISA technique following the manufacturer's guidelines (Demeditec, Germany). The following materials provided with the test kits were ready to use: antibody-coated wells, CRP conjugate reagent, TMB reagent, stop solution, hs-CRP sample diluent, and reference standard set. A standard curve of optical density was produced for each calibration standard with the hs-CRP ELISA kit and corresponding concentration values (mg/L). High levels of CRP (>3.0 mg/L) are considered to be high-risk.

#### 3.6.3. The Quantitative Determination of Ferritin

ELISA was used to measure circulating serum ferritin levels following the manufacturer's guidelines (Pointe Scientific, Inc., USA). Regarding the corresponding ferritin concentration values that were displayed in terms of ng/ml, the ferritin cut-off value was taken as 20 ng/ml in adult men and 10 ng/ml in adult women.

#### 3.6.4. Statistical Analysis

Data were analyzed using SPSS statistical software version 24.0, (SPSS Inc., Chicago, IL, USA). The normality test revealed that our data were abnormally distributed; hence, we considered the nonparametric Mann–Whitney *U* test for comparison between two groups (severe 1 pneumonia with O2 and severe 2 pneumonia without O2); variables with and without normal distribution were compared with Students' unpaired two-sided *t*-test and the Mann–Whitney *U* test. The results in this study are expressed as mean ± standard deviation (SD) while the comparison between multiple groups (total severe, mild, and healthy groups) was tested using the Kruskal–Wallis one-way analysis of variance and ANOVA one-way. The analysis of the odds ratio (OR) for risk factors of COVID-19 was performed using logistic regression. The Pearson correlation coefficient was employed to see whether there were any linear correlations between the two variables. For all analyses of tests, *p* values of 0.05 were considered significant.

## 4. Results

**Figure 1:**
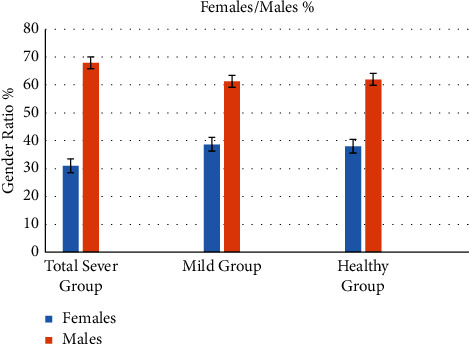
The percentages of males and females for the three groups.

It is apparent from Table 1 that the three major groups categorized in the study, total severe infected patients, mildly infected patients, and healthy individuals, had no significant differences (*p* < 0.05) in the characteristics such as sex distribution (gender ratio), age range, weight, and BMI. Furthermore, the individuals of all groups had no comorbidities, chronic diseases such as asthma and allergic diseases, or any other inflammatory diseases as managed by specific excluded criteria.  Figure 1 refers to the percentages of males and females for the three groups.

Data analysis to compare the hematological parameters of the groups revealed that there were significant differences between the healthy group and each of the severe and mild groups (*p* > 0.05), as well as there was a significant difference between the total severe and mild groups (*p* > 0.05) in WBC and neutrophils count. While the differences in the lymphocytes count among the groups appeared in different ways, we found a significant decline in lymphocytes count of the total severe group (1.0 ± 0.74 10^9^/L) compared to both mild (3.05 ± 0.67 10^9^/L) and healthy groups (3.01 ± 0.65 10^9^/L), and there was no significant difference between the mild and healthy groups, as seen in Table 2. Regarding monocytes count, it was a significant difference between every two groups of comparison (*p* > 0.05). The mean values of RBC showed a significant decline in the total severe group compared to the mild and the healthy groups (3.12 ± 0.78 versus 4.67 ± 1.23 10^12^/L and 3.12 ± 0.78 versus 4.86 ± 0.48 10^12^/L) (*p* > 0.05). But no significant difference was found between the two severe groups as seen in Table 3. The same profile was found regarding Hb. The mean value of Hb was significantly decreased in the total severe group compared to the other groups (10.68 ± 2.05 versus 11.84 ± 2.61 and 13.04 ± 1.34 g/dl, respectively) (*p* > 0.05), and there was no significant change between the two severe groups.

Regarding inflammatory parameters as seen in Table 2, both ESR and hs-CRP revealed a significant change between every two groups of comparison, with a more pronounced increase (*p* > 0.05), in the severe group (51.15 ± 24.4 mm/h and 42.97 ± 5.31 mg/l) for ESR and hs-CRP, respectively. The table illustrated that IL-6 was significantly elevated in the total severe group compared to the other two groups (660.4 ± 60.12 pg/ml) (*p*=0.0001). There was no significant difference between the mild and the healthy groups.

Analysis of biochemical data revealed that each of LDH, ferritin, ALT, and AST had significant differences when comparing the total severe group with every other group, and it was a clear elevation in the values of these parameters in the severe group (*p*=0.0001). On the other hand, there were no significant differences in these parameters between the mild and healthy groups. The same profiles of differences were found regarding each urea, albumin, D. bilirubin, and glucose which revealed significant changes between the total severe group and each of the other groups. No significant changes in D-dimer and APTT were found and Troponin T (ng/ml) was negative in each group of the study, as illustrated in Table 2.

Table 3 shows the comparison between the two subgroups of the total severe group patients (pneumonic with O2 requirement and pneumonic without O2 requirement) to reveal which parameter was highly affected due to the severity of the infection. It is apparent from the table that there were significant differences (*p* > 0.05) in the hematological profiles between pneumonic with O2 requirement and pneumonic without O2 requirement. Each of the WBC and neutrophils count increased significantly. Each lymphocyte and monocytes count was highly significantly affected by increasing the severity of the disease unlike the mean value of RBC which seemed nonsignificantly changed, as well as Hb (g/dl) was not significantly changed. Regarding the inflammatory parameters, there were significant changes represented by a highly significant effect in IL-6 (911.97 ± 40.94 versus 271.19 ± 16.75 pg/ml), ESR (55.59 ± 14.19 versus 44.11 ± 11.85 mm/h), and hs-CRP (46.29 ± 5.71 versus 37.70 ± 3.23 mg/L), (*p* > 0.05). Moreover, we found several variations in the biochemical parameters between the two subgroups as seen in Table 3. There were significant changes in each of urea (mg/dl), glucose (g/dl), and D-dimer (ng/ml) (*p* > 0.05) whereas there were no significant changes in each of LDH (U/L), ferritin (ng/ml), AST (U/L), albumin (g/dl), D. bilirubin (mg/dl), T. bilirubin (mg/dl), and APTT (sec) (*p* > 0.05). Both the subgroups showed a negative result for Troponin T (ng/ml).

The NLR was compared among multiple groups: healthy, mild, total severe, and (severe 1 and severe 2) revealed significant variations (*p* < 0.05), with a significant elevation in total severe patients. Moreover, NLR was highly elevated in severe 1 who required O2 (24.54 ± 2.4 with a mean difference of 22.99 within a limited range of error (22.45–23.52) at confidence interval (CI) 95%, as seen in Table 4.

Logistic regression for WBC, neutrophils, lymphocytes, Hb, ESR, hs-CRP, IL-6, and LDH was analyzed to find out which factors are more likely to be linked with the exacerbation of the disease. As illustrated in Table 5, IL-6, lymphocytes, and neutrophils were possible risk factors for COVID-19 infection, as well as IL-6 might represent the strongest factor associated with COVID-19 with a high odds ratio (OR: 24.138, 95% CI: 8.437–30.65, *p* < 0.01), more than the odds ratio of lymphocyte count (OR: 18.647, 95% CI: 1.542–22.719, *p* < 0.01) as seen in Table 5.

The relationship among IL-6, lymphocytes, neutrophils, monocytes, and inflammatory factors was analyzed by the Pearson correlation analysis. Significant positive correlations were found between the serum level of IL-6 and other immune-inflammatory factors: neutrophils count (*r* = 0.435, *p*=0.001), hs-CRP (*r* = 0.992, *p*=0.0001), glucose (*r* = 0.379, *p*=0.01), and monocyte count (*r* = 0.500, *p*=0.001). Otherwise, it was inversely related to the lymphocyte count (*r* = −0.816, *p*=0.0001) as illustrated in Table 6.

Table 7 illustrates the relationship between lymphocytes and other parameters in severe COVID-19 patients, revealing that lymphocyte count was inversely correlated to WBC count (*r* = −0.755, *p*=0.001), neutrophil counts (*r* = −0.543, *p*=0.002), and D-dimer (*r* = −0.430, *p*=0.004). The more surprising correlation is with Hb; interestingly, this correlation is positively related to lymphocyte count (*r* = 0.325, *p*=0.03). As shown in Table 8, there was a significant correlation among the indicators. A distinctive result was found as a significant positive relationship between LDH and the neutrophils count (*r* = 0.328, *p*=0.05).

## 5. Discussion

Although it was well established that COVID-19 could result in tissue damage, specifically in severe cases of infections, more studies are required to fight against the pandemic and any new study may be workable even with previously studied parameters. Tissue damage caused by COVID-19 could be detected by several clinical indicators including hematological, immunological, and biochemical markers. We investigated the clinical indicators associated with the damage caused by infection with the virus in three types (mild, severe with or without O2 requirement) of infection, depending on the severity, to reveal the most important predictor as a risk factor to develop the severity of the infection. Therefore, it was a comparative study between mild and the two forms of severe infection.

Starting with hematology tests, we found significant changes in the hematological parameters between healthy individuals and each of the severe and the mild groups. The findings of the present study showed that viral infection could produce various hematological changes. Each WBC count and neutrophil count were increased, while lymphocytes and RBC were decreased. This finding is consistent with findings of the past study by [34], which stated that the elevated WBC level might worsen and increase the risk of poor prognoses of the infection while the WBC count was not significantly changed in other studies [15]. However, the increased WBC count in our study was attributed to the increased neutrophils. The increase in the number of neutrophils was also found by another study [35], which stated that COVID-19 might be linked to the formation of the neutrophil extracellular traps (NETs), as in influenza virus infection, where the formed NETs lodge in and destruct pulmonary alveoli. Furthermore, the dysfunction of neutrophils was described as an important inflammatory marker that is involved in the pathological processes of certain viral infections like SARS [36]. Another study found higher neutrophils level in severe cases compared to mild [37]. Respiratory distress syndrome and lung injury in severe infection were associated with increased neutrophil filtration in the lung and elevated levels of neutrophils in the peripheral blood [38]. The significant decline in RBC in our study could be attributed to the effect of inflammation. Inflammatory processes might affect erythropoiesis through various mechanisms which finally lead to anemia of inflammation [39]. One of these mechanisms is abnormal according to the metabolism of iron which is due to the production of IL-6 and inflammatory cytokines such as IL-1, IL-33, and tumor necrosis factors. These factors inhibit the precursor cells of erythropoiesis and reduce the lifespan of erythrocytes [40]. We found a significant decline in lymphocyte count in the severe group compared to both mild and healthy groups, which was consistent with the finding of other investigations [41, 42]. There are multiple mechanisms mentioned that may act concomitantly to cause lymphopenia in COVID-19 patients; for example, lymphopenia could be attributed to either the direct attack on the lymphocytes resulting in lymphocytic destruction as well as lymphocyte sequestration in the lung, suppression of hematopoietic stem cells, or indirect ways including cytokine storm that stimulates apoptosis of lymphocytes [43]. Lymphopenia may be considered a common observation in patients with severe acute respiratory syndrome (SARS) caused by the SARS virus, as stated by previous studies [44, 45]. According to our results, lymphocyte count may serve as a quick tool to identify COVID-19 patients with severe cases. The most striking result that emerged from the data is a strong negative correlation between each lymphocyte count with IL-6, neutrophils, and WBC cells. Interestingly, this correlation might explain the dynamic and pathophysiological changes in the numbers of immune cells. This is confirmed by a recent study of our results reported by [46].

Moreover, we found an increase in the neutrophil-lymphocyte ratio. This result is in agreement with what was concluded by a previous study [47]. Recently, it has been found that NLR is an additional indicator for severe infection with COVID-19 despite the fact that using the NLR in the diagnosis of pneumonia was not common [45]. Based on evidence from previous studies, severe infection with COVID-19 is characterized by increased neutrophils and WBC and decreased lymphocytes [46]. The change in the ratio of neutrophils to lymphocytes may work as an indicative marker of severe progression of inflammatory processes that results in the development of certain complications so far observed in COVID-19 [48].

Regarding inflammatory parameters, both ESR and hs-CRP revealed significant increases, in the total severe group when compared to mild and healthy groups. Moreover, there was a significant change in ESR between patients who required O2 supplements and those who did not require them. This result refers to an increase in the inflammatory processes with the severity of the diseases. The ESR may occur rapidly during inflammation due to an increase in the fibrinogen portion in the blood that makes RBC stick together [49]. We found the same finding related to hs-CRP; it revealed significant differences between the mild and the severely infected patients as well as it was a significant elevation in severe patients who required O2. This finding is consistent with the findings of the previous study [50]. It is worth mentioning that the hs-CRP level is significantly elevated in bacterial infections more than in viral infections [51]. The current study revealed significantly higher hs-CRP levels in severe than in mild patients. This finding suggests that the hs-CRP level may be a biomarker of disease severity and progression in patients with COVID-19. This result is in agreement with a previous study [52]. Thus, the hs-CRP level is correlated with the level of inflammation, and its concentration level is not affected by factors such as age, sex, and physical condition; this finding agrees with what was stated by another study [53]. High-sensitivity *C*-reactive protein can activate the complement and enhance phagocytosis, thus clearing the pathogens that may invade the body [54]. An increase in the level of blood CRP may act as a nonspecific inflammatory indicator and play an instinctive role in the immune response [55]. It has been found that its level is associated with bronchitis and pneumonia [56]. Serum level of hs-CRP can be a rapid diagnosis of pneumonia [50]. It is released from hepatocytes in response to the stimulation of IL-6. It could serve as an indicator of a cytokine storm. Thus, increased levels of hs-CRP might be associated with the increased formation of inflammatory cytokines in severe patients with COVID-19. This was confirmed by our study through the Pearson correlation coefficient, with the existence of a strong positive correlation between IL-6 and hs-CRP, and this finding has consented with a previous study [57]. The normal level of CRP is ranging between 0.3 and 1.0 mg/dl but the level above 20 mg/dl refers to the hyperinflammatory process [58]. Patients with a value greater than this value may experience the development of cytokines storm and activation of the immune system in a way that can damage pulmonary tissue [56, 59]. Data analysis revealed that the mean IL-6 level was more than three times higher in the severe group compared to the other two groups while there was no significant difference between the mild and the healthy. This result was in agreement with what was found by other studies [60], suggesting that IL-6 might play a distinctive role in the development of COVID-19 and it is associated with the severity of the infection. Besides its role in pneumonia, it was found that its concentration is increased in pulmonary impairment confirming its role in the pathogenesis of lung injury caused by COVID-19 [60]. The virus can enter the pulmonary immune cells by binding to specific receptors (angiotensin-converting enzyme 2) which stimulates pulmonary immune cells to release proinflammatory factors including IL-6.

These inflammatory factors can destroy pulmonary tissue resulting in respiratory failure. It is well known that IL-6 is a many-sided factor with an important function in regulating the immune-inflammatory responses and stimulating the inflammatory processes and cytokine storm [61]. The level of IL-6 in a normal healthy individual is 0–7 pg/ml. It may reach 25 pg/ml and above due to very severe viral infection; therefore, its clinical management was used to diagnose infectious diseases [62]. This fact confirms what we found in this study by investigating the correlation between IL-6 and other inflammatory parameters as represented in Table 5. There was an inverse association between serum IL-6 concentrations and absolute blood count of lymphocytes in COVID-19 patients, a finding consistent with the results of other studies [63]. Therefore, it seems that IL-6 may play a role in lymphopenia that occurred in COVID-19 patients. Cytokine storm caused by an increased secretion of proinflammatory cytokines and chemokines may induce apoptosis of lymphocytes [64].

Regarding the biochemical parameters, ferritin revealed a significant increase in patients with severe infection. The increased level is correlated to the severity of COVID-19 infection. It is considered a marker of both inflammatory processes and dysregulation of immune function. Ferritin synthesis occurs intracellularly to bind with the iron storage inside the cells. However, serum ferritin is considered to be one of the earliest markers of inflammation and cell injury because it only occurs outside the cells in case of rupture of damaged cells. An increase in the formation of ferritin gives a larger binding capacity for intracellular iron which is a defense mechanism of the host immune system to minimize reactive oxygen species production inside the neutrophils and macrophages. Ferritin excites the damaged cell leaving behind the unbounded iron which results in further damage to the cell. Red blood cells are highly exposed to that damaging effect due to the high ferritin level inside them [65, 66]. Another biochemical factor that showed a significant increase in severely infected patients is LDH; this is supported by a previous study that revealed that LDH and hs-CRP may be related to respiratory function and be a predictor of respiratory failure in COVID-19 patients. LDH is an enzyme that is associated with metabolic acidosis; therefore, it is considered a marker of cytokine storm caused by COVID-19 infection [67]. Tissue hypoxia due to the viral infection results in metabolic acidosis that is caused by the formation and accumulation of lactic acid. Lactic acid stimulates the monocytes to produce inflammatory markers and increases the inflammatory processes as a result; LDH is increased as a response to homeostasis. Therefore, LDH is a workable indicator of the progression of the infection. However, our result is matching with the result of other studies [68]. One of the interesting findings of our study was the positive correlation between LDH and neutrophils count. As seen in Table 2, both ALT and AST were increased significantly in total severe infected patients when compared to the mild and healthy groups, but we did not find such significant differences in AST between the two severe cases. These enzymes are found in the hepatocytes and their secretion into the blood is increased in case of liver damage. It has been found that liver damage is among the clinical signs that characterize coronavirus patients with severe infection [69, 70]. Viral attack on the hepatocyte leads to an increase in these enzymes. On the other hand, we did not find significance between severe and mild infection in *D*-dimer, although *D*-dimer is an effective marker that indicates the thromboembolism process. Its level increases in case of activation of coagulation [64]. There were no significant changes in each of APTT, T. Bilirubin, and Troponin T. As seen in Table 5, lymphocytes and IL-6 might be considered risk factors to develop severe disease of the infection according to the odds ratio (IL-6: 24.138 and lymphocytes 18.647 at 95% of the confidence interval).

From the results and explanation above, some consistencies with other previous studies were observed; nevertheless, some differences could be attributed to several factors represented by sample size, sampling at a different period of the disease, and regional factors. It is worth mentioning that it is an accredited study; all participants were matching in age, sex, and free of comorbidities. However, this is a comprehensive study that revealed the role of each hematological, immunological, inflammatory marker, and biochemical indicator, which acts as an effective protagonist in diagnosing COVID-19, unlike previous studies that concentrated on certain parameters.

## 6. Conclusion

We conclude that the development of COVID-19 to the severe case is done by a complex network of factors that are somehow linked to each other as we found when analyzing the interactions between the factors. There were inverse effects on different hematological, immunological, and biochemical parameters which resulted from increased production of inflammatory markers such as IL-6 which plays a main role in inducing an inflammatory response, and a pathophysiology process which is known as the cytokine storm. As a result, each interleukin-6, lymphocytes, and neutrophils may be considered as major factors that affect the severity of COVID-19 through an inverse correlation.

## Figures and Tables

**Table 1:** Clinical characteristics and demographic of COVID-19 patients and healthy group.

Parameter	Total COVID-19 patients		Healthy	^*∗*^ *p*≤^*∗*^	1 vs 2	1 vs 3	2 vs 3
	Severe patients	Mild patients					
No of patients	88	62	50	—	—	—	—

*Gender ratio*							
Females (%)	28 (31)	24 (38.7)	19 (38)	NS	NS	NS	NS
Males (%)	60 (68)	38 (61.29)	31 (62)	NS	NS	NS	NS
Age (mean ± SD)	46.0 ± 11.07	40.37 ± 8.87	38.85 ± 6.87	NS	NS	NS	NS
Range (years)	(34–72)	(25–61)	(25–65)				
Weight (kg)	83.27 ± 3.06	89.12 ± 2.07	84.68 ± 2.04	NS	NS	NS	NS
BMI (kg/m^2^)	28.2 ± 7.82	30.41 ± 5.91	28.67 ± 6.56	NS	NS	NS	NS

*Comorbidities disease*							
Hypertension	No						
Diabetes mellitus	No						
Hyperlipidemia	No						
Other diseases	No						

^*∗*^ *p* is significant statistics at level <0.05. BMI: body mass index; NS: no significant.

**Table 2:** Comparative analysis of immunological, inflammatory, hematological, and clinical biochemical markers in COVID-19 patients according to their disease form.

Parameter	Total COVID-19 patients		Control (50)	^*∗*^ *p*≤^*∗*^	1 vs 2	1 vs 3	2 vs 3
	Severe (88)	Mild (62)					
WBC (10^9^/L)	13.60 ± 5.52	12.69 ± 4.79	7.34 ± 1.95	0.001	NS	0.001	0.001
Lym (10^9^/L)	1.0 ± 0.74	3.05 ± 0.67	3.01 ± 0.65	0.001	0.0001	0.0001	NS
Neu (10^9^/L)	11.34 ± 5.52	10.18 ± 4.02	4.50 ± 1.45	0.001	NS	0.0001	0.0001
Mon (10^9^/L)	0.99 ± 0.42	0.74 ± 0.42	0.43 ± 0.21	0.001	0.003	0.001	0.001
RBC (10^12^/L)	3.12 ± 0.78	4.67 ± 1.23	4.86 ± 0.48	0.006	0.002	0.001	NS
Hb (g/dl)	10.68 ± 2.05	11.84 ± 2.61	13.04 ± 1.34	0.04	0.02	0.003	0.001
ESR (mm/h)	51.15 ± 24.4	27.62 ± 12.97	8.17 ± 3.95	0.001	0.0001	0.0001	0.001
Hs-CRP (mg/L)	42.97 ± 5.31	14.17 ± 1.87	1.16 ± 0.88	0.0001	0.0001	0.0001	0.01
IL-6 (pg/ml)	660.4 ± 60.12	5.38 ± 0.37	4.54 ± 0.22	0.0001	0.0001	0.0001	NS
LDH (U/L)	672.40 ± 51.89	154.27 ± 22.5	157.2 ± 22.3	0.0001	0.0001	0.0001	NS
Ferritin (ng/ml)	989.56 ± 100.2	77.20 ± 3.31	76.02 ± 3.56	0.0001	0.0001	0.0001	NS
ALT (U/L)	33.01 ± 3.6	16.28 ± 1.02	16.05 ± 1.14	0.0001	0.002	0.002	NS
AST (U/L)	37.31 ± 3.02	14.17 ± 1.13	12.91 ± 0.84	0.0001	0.001	0.001	NS
Creatinine (mg/dl)	1.17 ± 0.33	0.68 ± 0.03	0.65 ± 0.13	0.1	NS	NS	NS
Urea (mg/dl)	65.78 ± 7.27	29.93 ± 9.06	27.91 ± 6.5	0.001	0.001	0.001	NS
Albumin (g/dl)	3.37 ± 0.47	4.26 ± 0.45	4.34 ± 0.40	0.001	0.01	0.01	NS
Direct bilirubin (mg/dl)	0.18 ± 0.02	0.29 ± 0.14	0.28 ± 0.13	0.002	0.001	0.005	NS
Total bilirubin (mg/dl)	0.62 ± 0.05	0.68 ± 0.04	0.67 ± 0.04	0.6	NS	NS	NS
Glucose (g/dl)	217.1 ± 38.9	97.43 ± 17.4	93.42 ± 11.6	0.001	0.0001	0.0001	NS
D-dimer (ng/ml)	2.66 ± 0.42	2.62 ± 0.40	2.18 ± 0.04	0.5	NS	NS	NS
APTT (sec)	28.38 ± 1.10	35.75 ± 6.13	37.02 ± 7.3	0.4	NS	NS	NS
Troponin T (ng/ml)	Negative	Negative	Negative	—	—	—	—

^*∗*^ *p* value is significant statistics at level <0.05. ^*∗∗*^LSD was used to determine multiple comparisons among three groups (post hoc tests). The data is presented as mean ± standard deviation (range). WBC: white blood cells. Lym: lymphocytes. Neu: neutrophils. Mon: monocytes; Hb: hemoglobin; hs-CRP: highly sensitive C-reactive protein; LDH: lactate dehydrogenase; APTT: activated partial thromboplastin time; NS: no significant; RBC: red blood cells; IL-6: interleukin-6; AST: aspartate aminotransferase; ALT: alanine aminotransferase.

**Table 3:** Comparative analysis of immunological, inflammatory markers, and hematological and clinical biochemical characteristics between two groups of total severe patients with and without O2 requirement.

Parameter	Total COVID-19 patients (No. = 88)		^*∗*^ *p*≤^*∗*^
	Severe patients (pneumonia with O2 requirement)	Severe patients (pneumonia without O2 requirement)	
No of patients	**54 (61%)**	**34 (39%)**	—
Sex, F/M	**14/40**	**14/20**	**0.02**
Age (years)	**43.6** **±** **8.82**	**45.70** **±** **13.09**	NS
Range	**(34–72)**	**(34–68)**	
WBC (10^9^/L)	**14.51** **±** **5.70**	**12.15** **±** **4.86**	**0.04**
Lym (10^9^/L)	**0.544** **±** **0.02**	**1.72** **±** **0.11**	0.0001
Neu (10^9^/L)	**12.27** **±** **0.82**	**9.86** **±** **0.69**	0.04
Mon (10^9^/L)	**0.59** **±** **0.04**	**0.97** **±** **0.07**	0.0001
RBC (10^12^/L)	**3.93** **±** **0.6**	**4.59** **±** **1.1**	NS
Hb (g/dl)	**10.94** **±** **2.59**	**11.15** **±** **1.43**	NS
ESR (mm/h)	**55.59** **±** **14.19**	**44.11** **±** **11.85**	**0.03**
Hs-CRP (mg/L)	**46.29** **±** **5.71**	**37.70** **±** **3.23**	**0.009**
IL-6 (pg/ml)	**911.97** **±** **40.94**	**271.19** **±** **16.75**	**0.0001**
LDH (U/L)	**713.50** **±** **51.53**	**607.11** **±** **45.79**	NS
Ferritin (ng/ml)	**1010.64** **±** **101.96**	**976.29** **±** **95.93**	NS
ALT (U/L)	**35.84** **±** **5.34**	**28.51** **±** **3.61**	**0.02**
AST (U/L)	**35.48** **±** **2.29**	**40.20** **±** **4.10**	NS
Creatinine (mg/dl)	**1.45** **±** **0.37**	**0.740** **±** **0.07**	**0.01**
Urea (mg/dl)	**76.85** **±** **7.41**	**48.20** **±** **4.75**	**0.006**
Albumin (g/dl)	**3.40** **±** **0.50**	**3.33** **±** **0.41**	NS
Direct bilirubin (mg/dl)	**0.18** **±** **0.01**	**0.20** **±** **0.03**	NS
Total bilirubin (mg/dl)	**0.67** **±** **0.04**	**0.55** **±** **0.05**	NS
Glucose (g/dl)	**241.49** **±** **16.27**	**178.36** **±** **18.29**	**0.01**
D-dimer (ng/ml)	**2.13** **±** **0.07**	**3.51** **±** **0.70**	**0.01**
APTT (sec)	**29.0** **±** **9.22**	**27.41** **±** **1.70**	NS
Troponin T (ng/ml)	Negative	Negative	

^*∗*^ *p* denotes statistical significance at the 0.05 level. Data are presented as mean ± standard deviation (range).

**Table 4:** Comparison of NLR among total severe, mild patients, and healthy control.

Group study	NLR Mean ± SD	*p*≤^*∗*^	Mean difference	95% CI of the difference	
				Lower	Upper
Total severe patients	24.54 ± 2.4	0.0001	22.99	22.45	23.52
Mild patients	4.79 ± 1.2	0.0001	3.24	2.97	3.51
Severe 1 patients	24.54 ± 2.4	0.0001	22.99	22.45	23.52
Severe 2 patients	3.02 ± 1.30	0.0001	1.47	1.18	1.76
Healthy control	1.55 ± 0.56	0.0001	Cut-off	—	—

^*∗*^ *p* value is significant statistics at level <0.05. Data are presented as mean ± standard deviation. NLR: percentage of neutrophil lymphocytes; CI: confidence interval.

**Table 5:** Odds ratio and confidence interval for risk factors of COVID-19 infection.

Factor	Odd ratio^*∗*^	95% confidence interval		*p*≤^*∗*^
		Lower	Upper	
White blood cells (WBC 10^9^/L)	2.37	0.57	9.70	0.01
Neutrophils (neu 10^9^/L)	3.121	0.60	16.09	0.02
Lymphocytes (lym 10^9^/L)	18.647	1.54	22.71	0.001
Hemoglobin (hb (g/dl))	0.791	0.04	13.12	NS
High-sensitivity C-reactive protein (hs-CRP)	0.248	0.024	2.49	NS
Erythrocyte sedimentation rate (ESR mm/h)	0.432	0.12	1.46	NS
Interleukin-6 (pg/ml)	24.138	8.43	30.65	0.0001
Lactate dehydrogenase (LDH) (U/L)	0.795	0.04	13.17	NS

^*∗*^ *p*value is significant statistics at level <0.05.

**Table 6:** Relationship between IL-6 and each lymphocyte, monocyte, neutrophil, hs-CRP, and glucose among severe COVID-19 patients.

Correlation		Lym	Mon	Neu	Hs-CRP	Glucose
IL-6	*r*	−0.816^*∗∗*^	0.500^*∗∗*^	0.435^*∗∗*^	0.992^*∗∗*^	0.379^*∗*^
	*P*	0.0001	0.001	0.001	0.0001	0.01

^*∗∗*^Correlation is significant at the 0.01 level (2-tailed). ^*∗*^Correlation is significant at 0.01 (two-tailed).

**Table 7:** Correlation among lymphocytes and each immune cell and other inflammatory factors in COVID-19 patients with severe disease.

Correlation		Mon	WBC	Neu	Hb	Glucose	D-dimer
Lymphocyte	*r*	−0.411^*∗∗*^	−0.755^*∗∗*^	−0.543	0.325^*∗*^	−0.324^*∗*^	−0.430^*∗∗*^
	*p*	0.006	0.001	0.002	0.03	0.03	0.004

**Table 8:** Estimation of the correlation between various factors depends on the study.

Correlation	WBC & Neu	RBC & Hb	ESR & hs-CRP	hs-CRP & Neu	LDH & Neu
*r*	0.884^*∗∗*^	0.821^*∗∗*^	0.310^*∗*^	0.351^*∗*^	0.328^*∗*^
*P*	0.0001	0.0001	0.04	0.01	0.03

## Data Availability

No data were used to support this study.
